# Different residues in the SARS-CoV spike protein determine cleavage and activation by the host cell protease TMPRSS2

**DOI:** 10.1371/journal.pone.0179177

**Published:** 2017-06-21

**Authors:** Lennart Michel Reinke, Martin Spiegel, Teresa Plegge, Anika Hartleib, Inga Nehlmeier, Stefanie Gierer, Markus Hoffmann, Heike Hofmann-Winkler, Michael Winkler, Stefan Pöhlmann

**Affiliations:** 1 Abteilung Infektionsbiologie, Deutsches Primatenzentrum, Göttingen, Germany; 2 Institut für Mikrobiologie und Virologie, Medizinische Hochschule Brandenburg Theodor Fontane, Senftenberg, Germany; New York Blood Center, UNITED STATES

## Abstract

The spike (S) protein of severe acute respiratory syndrome coronavirus (SARS-CoV) mediates viral entry into target cells. Cleavage and activation of SARS S by a host cell protease is essential for infectious viral entry and the responsible enzymes are potential targets for antiviral intervention. The type II transmembrane serine protease TMPRSS2 cleaves and activates SARS S in cell culture and potentially also in the infected host. Here, we investigated which determinants in SARS S control cleavage and activation by TMPRSS2. We found that SARS S residue R667, a previously identified trypsin cleavage site, is also required for S protein cleavage by TMPRSS2. The cleavage fragments produced by trypsin and TMPRSS2 differed in their decoration with N-glycans, suggesting that these proteases cleave different SARS S glycoforms. Although R667 was required for SARS S cleavage by TMPRSS2, this residue was dispensable for TMPRSS2-mediated S protein activation. Conversely, residue R797, previously reported to be required for SARS S activation by trypsin, was dispensable for S protein cleavage but required for S protein activation by TMPRSS2. Collectively, these results show that different residues in SARS S control cleavage and activation by TMPRSS2, suggesting that these processes are more complex than initially appreciated.

## Introduction

The subfamily *Coronavirinae* within the family *Coronaviridae* comprises viruses that cause respiratory, neurological and intestinal symptoms in mammals and birds. The severe acute respiratory syndrome coronavirus (SARS-CoV), a member of the genus Betacoronavirus, emerged in 2002 in Southern China. Its subsequent spread, mainly in Asia, wrecked massive economic havoc and was associated with almost 800 deaths [1,2]. Another betacoronavirus, the Middle East respiratory syndrome coronavirus (MERS-CoV), emerged in Saudi Arabia in 2012 and causes a SARS-like disease, particularly in patients with underlying chronic illness [3,4], indicating that emerging coronaviruses can constitute a severe threat to human health.

The SARS-CoV spike protein (SARS S) is incorporated into the viral envelope and mediates viral entry into target cells [5,6]. For this, the surface unit (S1) of SARS S binds to the cellular receptor angiotensin converting enzyme 2 (ACE2) [7,8] and the transmembrane unit (S2) then fuses the viral membrane with a host cell membrane. Activation of SARS S by a cellular protease is a prerequisite to membrane fusion and protease choice determines at which cellular localization membrane fusion occurs [9,10]. Thus, binding of SARS-CoV to ACE2 can trigger uptake of virions into host cell endosomes, where the pH-dependent cysteine proteases cathepsin B/L cleave and activate the S protein [11]. Alternatively, the type II transmembrane serine protease TMPRSS2 can cleave SARS S, potentially at or near the cell surface, which renders SARS S-driven entry independent of cathepsin B/L activity [12–14]. While both cathepsin B/L and TMPRSS2 can cleave and activate SARS S in cell lines, only TMPRSS2 activity seems to be required for viral spread in the host, since a serine protease inhibitor active against TMPRSS2 but not a cysteine protease inhibitor active against cathepsin B/L protected rodents from SARS-CoV-induced pathogenesis [15]. Such a scenario would be in keeping with the findings that TMPRSS2 expression is required for spread of several influenza A viruses (FLUAV) in mice [16–18] and that polymorphisms in TMPRSS2 impact severity of influenza in human patients [19].

It is at present not fully understood which sites in SARS S are cleaved by cellular proteases for S protein activation. Treatment of cell-associated, SARS S-bearing particles with trypsin, which is frequently used as a substitute for other S protein activating cellular proteases, can allow for cathepsin B/L independent entry [11] and SARS S residue R667 is essential for this process [20]. Similarly, human airway tryptase (HAT), a TMPRSS2-related enzyme, was shown to cleave SARS S at R667 and this residue was found to be critical for SARS S activation for cell-cell fusion [21]. R667 is located at the border between the S1 and the S2 subunit and defines the S1/S2 cleavage site. This site is found in diverse coronavirus S proteins, can be embedded in different cleavage motifs and is frequently cleaved during S protein biogenesis [9]. Moreover, a role of R797 in SARS S activation by trypsin was demonstrated [22]. Residue R797 defines the S2’ cleavage site, which is also present in other coronavirus S proteins, and is believed to be cleaved during viral entry [9]. However, cleavage of SARS S at this site could not be visualized so far, potentially due to the transient nature of the cleavage product. On the basis of these observations a two-step mechanism for SARS S activation was proposed, which encompasses consecutive cleavage of SARS S at R667 (S1/S2) and R797 (S2’) [22]. In contrast, it is at present unknown where SARS S is cleaved by TMPRSS2 for S protein activation, although it has been suggested that HAT and TMPRSS2 process SARS S at different sites [21].

Here, we investigated which residues in SARS S are required for cleavage and activation by TMPRSS2. We provide evidence that trypsin and TMPRSS2 produce SARS S fragments of identical amino acid sequences but with different N-glycan decoration, suggesting that these proteases cleave different SARS S glycoforms at the same site. In keeping with this finding, we demonstrate that R667 is required for SARS S cleavage by both trypsin and TMPRSS2 while R797 is dispensable. Conversely, R797 but not R667 is required for activation of SARS S by TMPRSS2.

## Material and methods

### Plasmid construction and in vitro mutagenesis

Expression plasmids for SARS S wt and mutant R667A have been described previously [20]. Plasmids encoding ACE2, TMPRSS2, vesicular stomatitis virus glycoprotein (VSV-G) and the MLV-based pseudotyping system have also been described previously [12,23,24]. Plasmids for expression of SARS S mutants K543A/R544A, R563A/K566A, T678S and R797N were generated by overlap-extension PCR. All SARS S constructs were equipped with a C-terminal V5 antigenic tag and were inserted into plasmid pCAGGS [25]. To obtain a plasmid encoding SYFP2-Mem, which was used as plasma membrane marker, we first replaced the EGFP gene in pEGFP-N1 (Clontech) by SYFP2 [26] and then transferred the N-terminal 20 codons of the neuromodulin gene from pEYFP-Mem (Clontech). Plasmid pE-GFP-Golgi, which encodes the Golgi marker beta-1,4-galactosyltransferase fused to GFP has been described previously [27]. The integrity of all PCR-amplified sequences was verified employing automated sequence analysis.

### Cell culture

Human embryonal kidney 293T cells and African green monkey derived COS-7 cells were grown in Dulbecco’s modified Eagle’s medium (DMEM) supplemented with 10% fetal bovine serum (FBS), penicillin, and streptomycin (1%) at 37°C in a humidified atmosphere containing 5% CO2. The identity of the 293T cell line was verified by short tandem repeat (STR) analysis. COS-7 cells were obtained from collaborators. All cells were tested negative for mycoplasma.

### Analysis of SARS S cleavage

For analysis of SARS S cleavage, plasmids encoding SARS S and TMPRSS2 or empty plasmid were transiently transfected into 293T cells employing the CaPO method. At 48 h post transfection that cells were either PBS treated or incubated with 50 μg/ml trypsin (Sigma-Aldrich, St Louis, USA) for 5–10 min at 37°C and then lysed in 2 x SDS sample buffer (0.03M Tris-HCl, 10% glycerol, 2% SDS, 5% beta-mercaptoethanol, 0.2% bromphenolblue, 1 mM EDTA) for 10 min at 95°C. Cell lysates were separated by SDS-PAGE and transferred onto nitrocellulose membranes (GE healthcare). Full length SARS S protein and cleavage fragments thereof containing the SARS S C-terminus were detected by staining with an anti-V5 antibody (Novex) and anti-mouse-HRP (Dianova) as secondary antibody. As loading control, the stripped membranes were incubated with an anti-β-actin antibody (Sigma-Aldrich) and anti-rabbit-HRP (Dianova) as secondary antibody.

### PNGase F digest

For analysis of SARS S modification with N-linked glycans, cells transfected to express SARS S in the presence or absence of TMPRSS2 or treated with trypsin were pelleted by centrifugation at 1,200 rpm for 5 min. Pellets were solved in TNE buffer (50 mM Tris-HCl, 0.15 M NaCl and 10 mM EDTA) and incubated for 1 h at 37°C with PNGase F (New-England Bio-Labs) according to the manufacturer’s instructions or control incubated in the absence of enzyme. Subsequently the samples were analyzed by immunoblotting as described above.

### Immunohistochemistry and confocal microscopy

COS-7 cells were seeded on glass coverslips in 24-well plates at 40,000 cells per well. 24 hours later, the cells were CaPO-cotransfected with plasmids encoding SARS S wt or mutants and marker proteins localized to the plasma membrane (N-terminal 20 amino acids of neuromodulin fused to YFP) or to the Golgi apparatus (β-1,4-galactosyltransferase fused to GFP) [27]. After 6 h of incubation at 37°C, cells were washed twice with PBS and fresh culture medium was added. At 48 h post transfection, cells were fixed and stained as described elsewhere [28] using mouse monoclonal IgG2a anti-V5 (Invitrogen) as primary antibody and Alexa Fluor 647 donkey anti-mouse IgG (Invitrogen) as secondary antibody. Fluorescence images were acquired and colored with the confocal laser scanning microscope LSM 5 Pascal Axioskop 2 MOT plus (Carl Zeiss Microimaging GmbH) using a 63x, NA1.4 Zeiss Plan Apochromat objective and LSM Pascal 5 software version 3. Confocal images were converted to JPEG format using the software AxioVision Rel. 4.8 (Carl Zeiss Microimaging GmbH).

### Analysis of SARS S activation

For the analysis of SARS S activation for virus-cell fusion, retroviral pseudotypes were employed, as described previously [24]. In brief, pseudotypes were produced by transient CaPO-transfection of 293T cells with plasmids encoding MLV-Gag-Pol, a luciferase encoding MLV vector and expression plasmids for SARS S wt and mutants. Cotransfection of plasmid encoding VSV-G served as positive control, cotransfection of empty plasmid served as negative control. The culture medium was replaced by fresh medium at 15 h and harvested at 40 h posttransfection. The supernatants were filtered through 0.45 μm pore-sized filters, aliquoted and stored at—80°C. The incorporation of glycoproteins into the MLV particles was examined by immunoblotting for MLV capsid protein (4B2, Acris) and SARS S as described above after concentration of pseudotypes by centrifugation through a 20% sucrose cushion.

For transduction experiments, 293T target cells were seeded in 6-well plates and transfected with plasmids encoding ACE2 and/or TMPRSS2. The culture medium was replaced at 6–8 h posttransfection and after 24 h cells were reseeded into 96-well plates. At 48 h posttransfection cells were incubated for 1 h at 37°C with 50 μM of the cathepsin B/L inhibitor MDL28170 (Sigma-Aldrich) or the same concentration of DMSO in a total volume of 50 μl per well. Subsequently, inhibitor was removed, 50 μl per well of pseudotype preparations were added to triplicate wells and 6 h thereafter medium was diluted (1:3). At 60 h post transduction cells were harvested and luciferase activities in cell lysates were measured employing a commercially available kit (PJK) according to the manufacturer’s instructions.

### Statistical analysis

A one-tailed student’s t-test was used to examine statistical significance.

## Results

### TMPRSS2 and trypsin cleave SARS S at the same site

**Fig 1:**
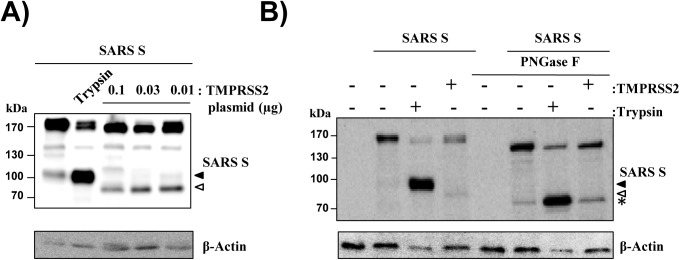
Trypsin and TMPRSS2 cleave SARS S at the same site but process different SARS S glycoforms. (A) 293 T cells were cotransfected with expression plasmids for SARS S and decreasing amounts TMPRSS2 encoding plasmid and treated with trypsin or PBS. Subsequently, SARS S cleavage was analyzed by Western blot employing anti-V5 antibody for S protein detection. Detection of β-actin served as loading control. Similar results were obtained in three independent experiments. The black filled triangle indicates the SARS S cleavage product generated by trypsin, while the product of SARS S processing by TMPRSS2 is marked by the white filled triangle. (B) The experiment was carried out as in (A). However, cells were treated with PNGaseF to remove all N-linked glycans before Western blot analysis. The asterisk indicates deglycosylated SARS S cleavage products. The results were confirmed in five independent experiments.

In order to identify SARS S residues required for S protein cleavage and activation by TMPRSS2, we first investigated which cleavage fragments are produced upon cotransfection of 293T cells with a constant amount of SARS S plasmid and decreasing amounts of TMPRSS2 plasmid, since expression of low amounts of protease in the transfected cells may best reflect TMPRSS2 expression in the lung. S protein with a V5 antigenic tag was employed for these studies and C-terminal S protein fragments were detected by Western blot employing a V5-specific antibody. This approach revealed the production of a SARS S fragment of 85 kDa by TMPRSS2 (Fig 1A). The intensity of the 85 kDa band increased upon reduction of TMPRSS2 expression, with the highest signals observed upon transfection of 0.03–0.01 μg TMPRSS2 plasmid (Fig 1A). In contrast, incubation of SARS S expressing cells with trypsin produced a 100 kDa fragment (Fig 1A), in keeping with published data [20]. We next investigated whether the different sizes of the SARS S cleavage fragments produced by TMPRSS2 and trypsin reflected differences in amino acid sequence or N-glycosylation status. Removal of N-glycans by PNGase F revealed that the latter was the case: The untreated SARS S fragments produced upon cleavage by trypsin and TMPRSS2 migrated with different speed in the gel while migration was identical upon PNGase F treatment (Fig 1B). These findings suggest that both trypsin and TMPRSS2 cleave SARS S at the same site but that SARS S isoforms exhibiting differential N-glycosylation are cleaved.

### R667 but not R797 is required for processing of SARS S by TMPRSS2 and trypsin

**Fig 2:**
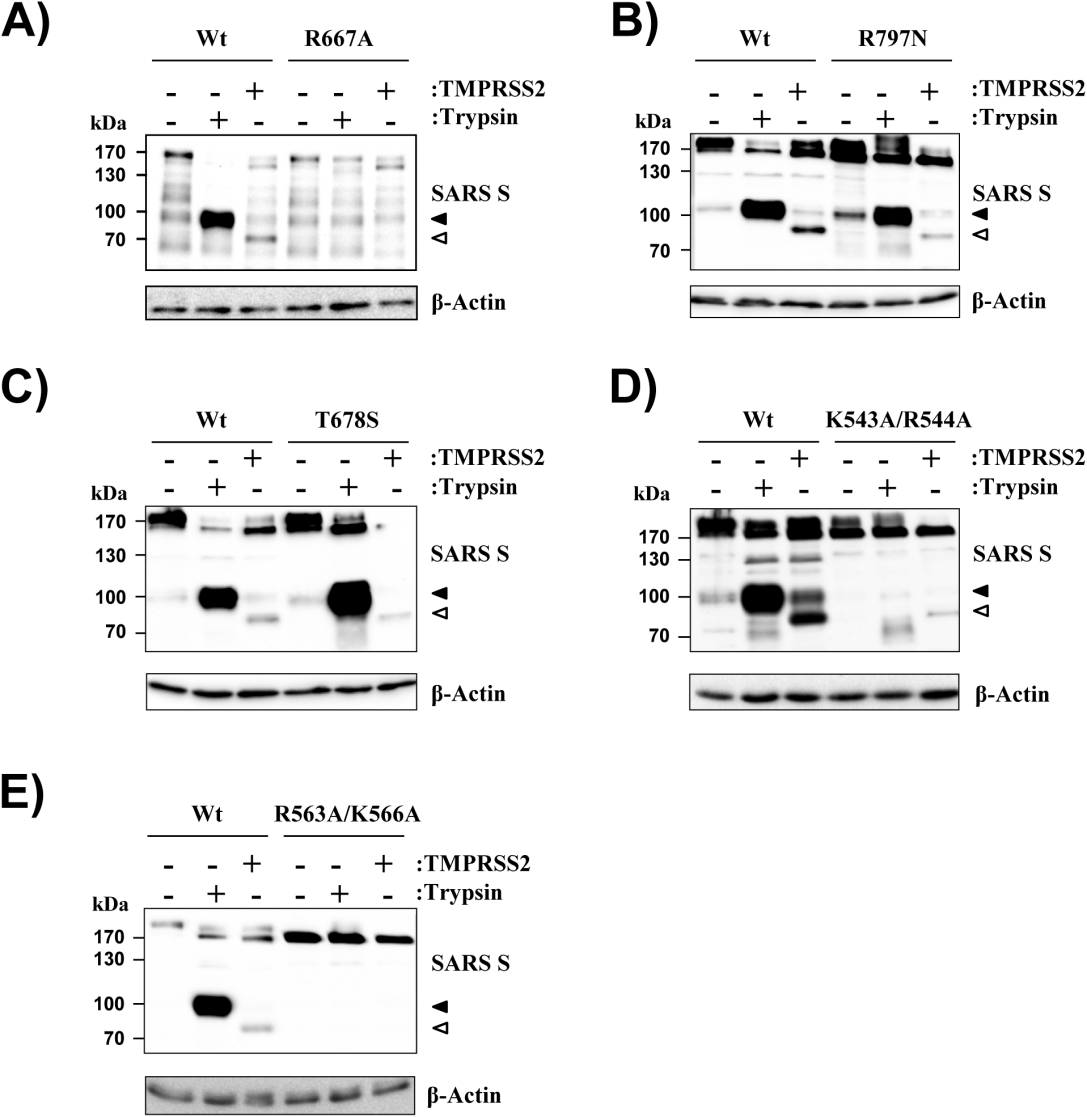
R667 but not R797 is required for SARS S cleavage by trypsin and TMPRSS2. Plasmids encoding SARS S wt or the indicated SARS S mutants were cotransfected with TMPRSS2 plasmid or empty plasmid into 293T cells. Subsequently, the cells were treated with trypsin or PBS and SARS S cleavage was analyzed by Western blot employing anti-V5 antibody. Detection of β-actin served as loading control. The results were confirmed in two to four separate experiments. The SARS S cleavage products generated by trypsin and TMPRSS2 are indicated by black and white filled triangles, respectively.

In order to identify the cleavage site(s) of TMPRSS2 in SARS S, we mutated residues previously reported to be required for SARS S cleavage and/or activation by trypsin, R667 and R797 [22,29,30]. Moreover, we mutated K543/R544 and R563/K566 in order to investigate whether potential cleavage motifs located N-terminal of R667 are involved in SARS S cleavage by TMPRSS2. Finally, we altered residue T678, which was previously suggested to be required for SARS S activation by cathepsin L [31]. All mutated residues are located at the surface of the S protein trimer and access by proteases is unlikely to be obstructed by N-glycans, with the potential exception of R563/K566 (S1 Fig). Mutation of R667 abrogated SARS S processing into the 100 kDa fragment by trypsin and the 85 kDa fragment by TMPRSS2, respectively (Fig 2A), indicating that both enzymes cleave SARS S at this site. In contrast, mutation of R797 did not inhibit SARS S processing by trypsin and had only a modest impact on S protein cleavage by TMPRSS2 (Fig 2B), and a similar observation was made for residue T678 (Fig 2C). Finally, mutation of K543/R544 inhibited SARS S processing by trypsin but was compatible with cleavage by TMPRSS2 (Fig 2D), although at reduced levels, while mutation of R563/K566 blocked SARS S cleavage by both trypsin and TMPRSS2 (Fig 2E). Collectively, these results indicate that residue R667 but not R797 or T678 is required for SARS S processing by TMPRSS2 and trypsin and suggest that also residues N-terminal of R667 might impact proteolytic processing of SARS S.

### Mutation of K543/R544 and R563/K566 interfere with SARS S trafficking

**Fig 3:**
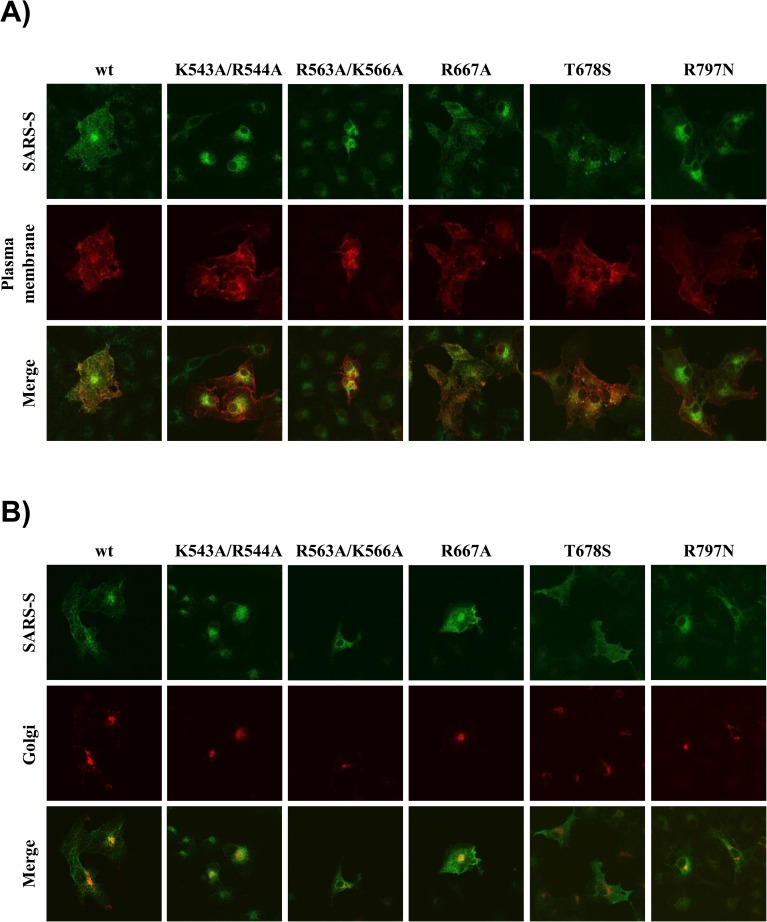
Mutations K543A/R544A and R563A/K566A interfere with SARS S trafficking through the constitutive secretory pathway. (A) COS-7 cells were cotransfected with expression plasmids for SARS S wt or the indicated mutants and with a plasmid encoding YFP fused to a plasma membrane anchor (red color). After staining for SARS S with V5 antibody and anti-mouse Alexa Fluor 647 secondary antibody (green color) the cells were analyzed by confocal microscopy. Similar results were obtained in a second independent experiment. (B) The experiment was carried out as described for panel A, but GFP fused to a Golgi marker (red color) was used instead of YFP with plasma membrane anchor. The results were confirmed in a second separate experiment. For optimal visualization, colors were manually assigned with LSM Pascal 5 software version 3 and do not correspond to the actual emission spectra of YFP, GFP and Alexa Fluor 647.

We next investigated whether differential cellular localization of the SARS S mutants might account for their differential processing by trypsin and/or TMPRSS2. Immunofluorescence of transfected COS-7 cells coexpressing SARS S wt and mutants jointly with YFP targeted to the plasma membrane revealed that SARS S wt and mutants R667A, T678S and R797N were trafficked to the cell surface, although intracellular levels of the mutant S proteins were slightly (mutants R667A, T678S) or markedly (R797N) higher than that observed for the wt protein (Fig 3A). In contrast, mutation of residues K543/R544 and R563/K566 resulted in intracellular retention of the mutant proteins (Fig 3A). In order to characterize the intracellular compartment in which mutants K543A/R544A and R563A/K566A accumulated, we repeated the experiment as described above but included a marker for Golgi localization instead of a plasma membrane marker. We found that the signals detected for mutant K543A/R544A partially overlapped with the Golgi signal while there was little overlap detected for mutant R563A/K566A (Fig 3B). However, only very few cells were detected that expressed high levels of mutant R563A/K566A, for at present unclear reasons. These results suggest that mutation of K543/R544 may render SARS S trypsin-resistant by precluding normal SARS S trafficking through the constitutive secretory pathway, which is required for S protein exposure to this protease. Similar reasons might account for the resistance of mutant R563A/K566A to cleavage by TMPRSS2 and trypsin.

### Mutation of residues R667, T678 and R797 is compatible with robust cell entry

Retroviral particles encoding a firefly luciferase reporter and harboring SARS S in their envelope (pseudotypes) are commonly used to study SARS S-driven host cell entry. Since we sought to employ pseudotyping to investigate the proteolytic activation of SARS S mutants, we next determined whether the mutations studied modulated SARS S incorporation into retroviral particles. These particles bud from the plasma membrane and one would thus expect that only S proteins expressed to appreciable levels at the cell surface are incorporated into particles.

**Fig 4:**
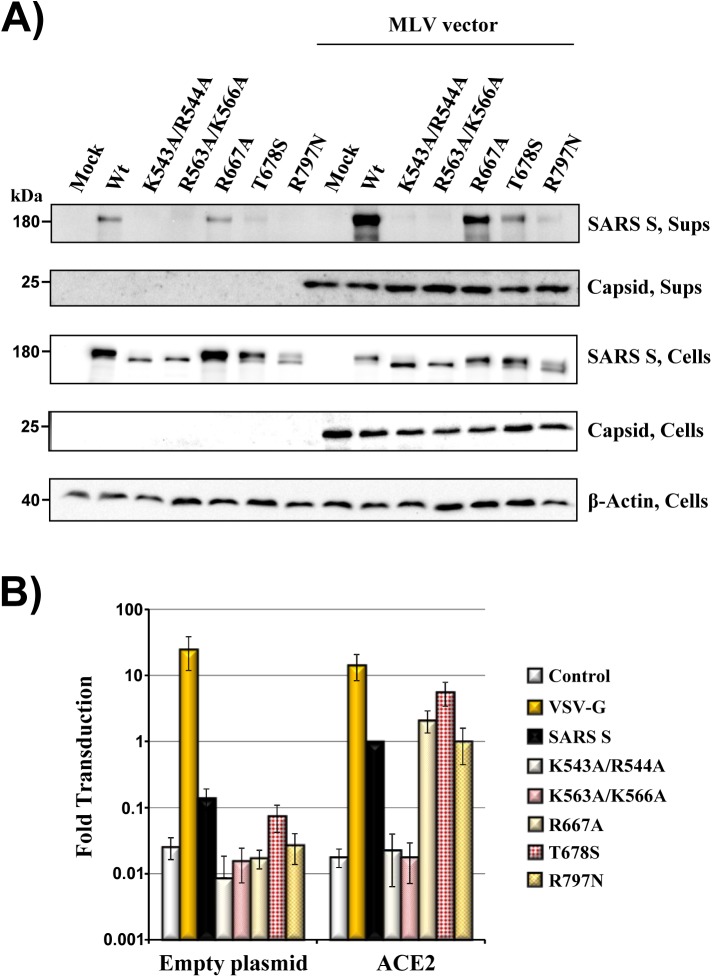
Mutations R667A, T678S and R797N are compatible with robust S protein-driven cell entry. (A) 293T cells transfected to express MLV particles pseudotyped with SARS S wt or the indicated SARS S mutants and the corresponding supernatants were analyzed for expression of S protein (employing anti-V5 antibody) and MLV capsid protein (employing anti-p30 antibody) by Western blot. Cells transfected to express S protein alone or transfected with empty plasmid were used as controls. Similar results were obtained in two independent experiments. (B) 293T target cells transfected with empty plasmid (control) or ACE2 plasmid were transduced with equal volumes of pseudotypes bearing the indicated viral glycoproteins. Transduction efficiency was quantified by measuring luciferase activities in cell lysates. Results were normalized for SARS S-driven transduction of ACE2 expressing cells, which was set as 1-fold. The average of three to eight independent experiments is shown. Error bars indicate standard error of the mean (SEM).

Analysis of particle preparations revealed robust and comparable signals for SARS S wt and mutant R667A, and inspection of control supernatants confirmed that these signals were mainly due to particle-associated S proteins (Fig 4A). Mutants T678S and R797N were detected in particles as well, although at reduced levels compared to SARS S wt, while mutants K543A/R544A and R563A/K566A were not incorporated into particles (Fig 4A), in keeping with their intracellular accumulation (Fig 3). In agreement with these findings, mutation of K543/R544 and R563/K566 was not compatible with particle infectivity (Fig 4B) while transduction driven by the other S protein mutants studied was roughly comparable to that measured for SARS wt, at least when 293T cells transfected to express ACE2 were examined (Fig 4B). Transduction of untransfected control cells (Fig 4B), which are known to express endogenous ACE2 at low levels [7], was generally less robust then transduction of ACE2-transfected cells and entry driven by most S protein mutants was reduced as compared to SARS S wt. These observations suggest that mutation of K543/R544 and R563/K566 interferes with normal S protein trafficking and incorporation into retroviral particles. In contrast, mutation of R667, T678, and R797 is compatible with S protein trafficking to the cell surface, particle incorporation and S protein-driven entry into cells expressing high levels of ACE2.

### R797 is required for SARS S activation by TMPRSS2

**Fig 5:**
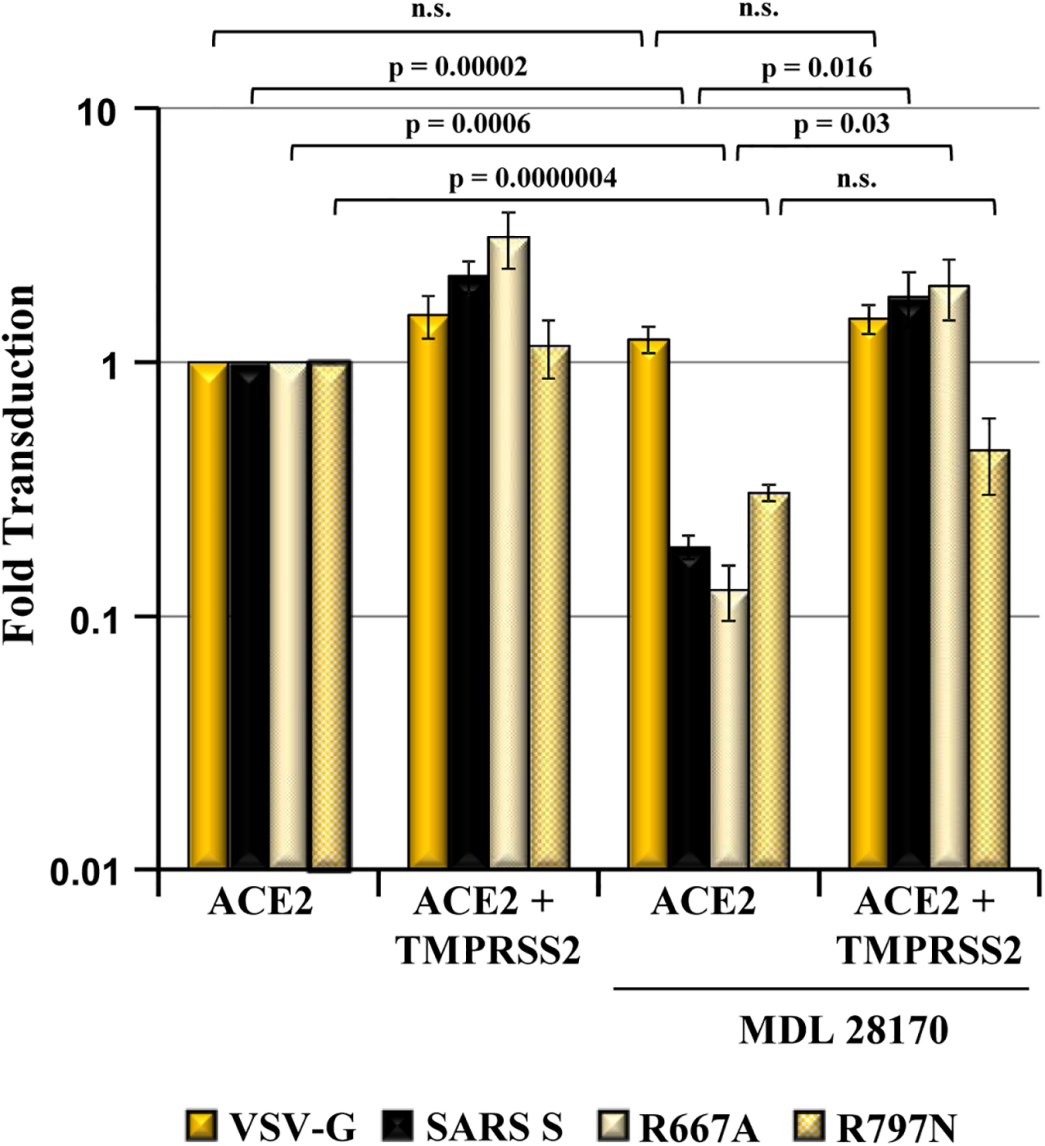
R797 but not R667 is required for SARS S activation by TMPRSS2. 293T target cells transfected with ACE2 plasmid or cotransfected with ACE2 and TMPRSS2 plasmid were pretreated with DMSO or the cathepsin B/L inhibitor MDL 28170 for 1 h before addition of equal volumes of pseudotypes bearing the indicated glycoproteins. Cellular entry was quantified by measurement of luciferase activity in cell lysates. Results were normalized for transduction of ACE2^+^, TMPRSS2^-^ cells in the absence of inhibitor, which was set as 1-fold. The average of three to six independent experiments is shown (R667A, n = 3; SARS S wt and VSV-G, n = 4; R797N, n = 6). Error bars indicate SEM. Statistical significance was assessed using one-tailed student’s t-est.

Finally, we assessed if amino acids R667 and R797 are required for SARS S activation by TMPRSS2. For this, we cotransfected 293T target cells with ACE2 plasmid and either empty plasmid or TMPRSS2 plasmid, and incubated the cells with DMSO or the cathepsin B/L inhibitor MDL 21870 before addition of SARS S-bearing particles. In the absence of inhibitor, TMPRSS2 expression modestly increased cellular entry driven by SARS S wt and mutant R667A but failed to augment entry driven by mutant R797N. Incubation of ACE2^+^,TMPRSS2^-^ cells with MDL 28170 significantly reduced transduction driven by SARS S wt as compared to untreated cells and this effect was rescued by expression of TMPRSS2 (Fig 5), as expected [12–14]. Similar results were obtained for mutant R667A, which was resistant to processing of SARS S by trypsin and TMPRSS2 (Fig 2). In contrast, entry into ACE2^+^, TMPRSS2^-^ cells mediated by mutant R797N was modestly (but significantly) blocked by MDL 21870 and this blockade was not rescued upon coexpression of TMPRSS2 (Fig 5), suggesting that R797 is critical for SARS S activation by TMPRSS2. Thus, R667 seems to be required for SARS S cleavage but not activation by TMPRSS2 while the reverse applies to residue R797.

## Discussion

Several recent findings suggest that TMPRSS2 is a potential target for antiviral intervention: Expression of this protease is essential for spread of several FLUAV strains in mice [16–18], TMPRSS2 polymorphisms which increase protease expression are linked with severe influenza in humans [19] and a serine protease inhibitor which blocks TMPRSS2 (and other serine proteases) protects rodents from SARS-CoV pathogenesis [15]. Here, we investigated which determinants in SARS S control cleavage and activation by TMPRSS2. Residue R667 was found to be essential for SARS S cleavage by TMPRSS2 while the same residue was dispensable for S protein activation. Conversely, residue R797 did not impact SARS S cleavability by TMPRSS2 but was essential for virus activation by this protease. In the following we will discuss and provide potential explanations for these at first sight paradoxical findings.

We previously documented that coexpression of high amounts of TMPRSS2 and SARS S results in the production of several C-terminal cleavage fragments [12]. In contrast, a single prominent cleavage product was observed in the present study. These differences are due to choice of antibodies used for SARS S detection: In our initial study we employed an S2-specific rabbit serum and thus detected all cleavage fragments harboring the antibody target irrespective of the presence of the SARS S C-terminus. In contrast, an antibody specific for the V5-tag added to the C-terminus of SARS S was used in the present study and thus only SARS S fragments with intact C-terminus were detected. The major cleavage product observed in the present study exhibited a molecular weight of 85 kDa and comprised the entire S2 subunit, as discussed below. The production of this fragment was increased when TMPRSS2 expression was decreased, suggesting that this fragment might be the major cleavage product generated under physiological conditions (i.e. when TMPRSS2 expression levels are low), although this effect could be partially due to previously documented cytotoxic effects observed upon directed TMPRSS2 expression in cells [14]. Two points should be considered when interpreting the results of our cleavage analysis: First, under the conditions chosen a substantial fraction of S proteins are secreted into the extracellular space due to TMPRSS2-mediated cleavage of SARS S at a membrane proximal site [12]. The production of most of these fragments remained undetected in the present study (due to usage of an antibody specific for the C-terminal V5 tag as discussed above) but most likely accounted for the relatively modest intensity of the signal observed for the membrane associated 85 kDa SARS-S fragment. Second, we employed a cis setting (SARS S and TMPRSS2 expressed in the same cells) to study SARS S cleavage. This setting allows to readily detect the production of SARS S cleavage fragments [12] but does not reflect that SARS S and TMPRSS2 are located in different membranes (i.e. viral and cellular membrane, respectively) during viral entry. Moreover, it needs to be considered that SARS S might be associated with other viral membrane proteins in the context of SARS-CoV infection, which are absent in the transfected cells examined here.

We reported previously that trypsin and TMPRSS2 cleave SARS S at different sites since these enzymes produced different SARS S cleavage fragments [21]. Differences in the size of the SARS S cleavage products generated by TMPRSS2 and trypsin were also observed in the present study: Trypsin generated a 100 kDa fragment while TMPRSS2 produced an 85 kDa fragment. However, enzymatic digest revealed that the 15 kDa difference in molecular weight resulted from differential decoration of these fragments with N-glycans and not from differences in the amino acid chain. In keeping with this finding, mutation of R667 inhibited SARS S cleavage by both trypsin and TMPRSS2, indicating that both proteases cleave SARS S at R667.

In contrast to the findings discussed above, our published work argued against a role of R667 in SARS S cleavage by TMPRSS2 [21]. Employing an S1 specific antibody, we had observed two prominent SARS S cleavage fragments upon coexpression of TMPRSS2 and production of both fragments was reduced but not abrogated upon mutation of R667 [21]. Since this reduction was modest, we then concluded that R667 is not essential for SARS S processing by TMPRSS2 [21]. In the light of the findings of the present study, we have to revise our conclusion: The previously observed reduced production of S protein fragments was due to mutation of R667 and more prominent effects were most likely missed because higher amounts of TMPRSS2 were expressed in the previous as compared to the present study.

Why does digestion of SARS S by TMPRSS2 and trypsin generate differentially glycosylated S protein fragments? Our results indicate that the differential glycosylation of the cleavage products might reflect differential cellular localization of the corresponding cleavage processes. Thus, trypsin (which is added to the culture medium) should cleave almost exclusively SARS S located at the cell surface. In contrast, TMPRSS2 might process SARS S early after import in the constitutive secretory pathway and before N-glycans are fully processed. Such a scenario is supported by the finding that TMPRSS2 is mainly localized within the cell and that activation of FLUAV hemagglutinin by TMPRSS2 occurs mainly intracellularly [32,33]. The potential impact of the cellular localization of SARS S and protease on S protein processing is also reflected by mutant K543A/R544A. This mutant is unable to fully traverse the constitutive secretory pathway and this defect is compatible with cleavage by TMPRSS2 (within the cell) but not trypsin (cleavage at the cell surface). Whether the postulated processing of SARS S by TMPRSS2 early during traversal of the secretory pathway is observed in the context of SARS-CoV infected human respiratory cells or is due to TMPRSS2 overexpression remains to be determined.

SARS S can be activated by TMPRSS2, which exhibits trypsin-like specificity, and trypsin can be employed to substitute the action of this protease. Previous work demonstrated that trypsin cleaves SARS S at R667 [20–22] and the present study shows that the same residue is critical for SARS S processing by TMPRSS2. Does the requirement of R667 for SARS S cleavage by trypsin and TMPRSS2 translate into a key role of this residue in SARS S activation for virus-cell fusion by these proteases? Previous studies showed that mutation of R667 abrogates SARS S activation by trypsin [20,22] while the present work demonstrates that this residue is dispensable for SARS S activation by TMPRSS2. Conversely, R797 is required for SARS S activation by trypsin [22] and TMPRSS2 (present study), although this residue is not essential for robust S protein processing by these proteases ([22] and present study), at least in the cis setting. It has been proposed that cleavage at R797 occurs in trans but a cleavage fragment could not be detected, potentially due to its low stability [22]. At present, it is unclear why cleavage and activation of SARS S by trypsin depend on the same residue, R667, while this is not the case for TMPRSS2. However, this finding is not entirely unexpected: Belouzard and Whittaker previously suggested that SARS S activation might require two consecutive cleavages, one at R667 and one at R797 but they also found that residue R797 is dispensable for cathepsin B/L-dependent, SARS S-driven endosomal entry [22]. Moreover, the present study shows that neither R667 nor T678 are required for SARS S-mediated endosomal entry, as evidenced by the robust transduction of untreated, ACE2-transfected 293T cells by SARS S mutants R667A and T678S (Fig 4B) and blockade of entry by MDL 28170 (R677A, Fig 5). These findings suggest that the relatively low sequence specificity of cathepsin B/L might allow for SARS S cleavage-activation at sites other than R667 and R797. It is conceivable that TMPRSS2 employs the same auxiliary cleavage site as cathepsin B/L when R667 is not available and the identification of this site should be in the focus of future studies.

Collectively, our study shows that SARS S cleavage and activation by TMPRSS2 depend on different amino acid residues, R667 and R797, and further work is required to elucidate their interplay in S protein activation for membrane fusion.

## Supporting information

S1 Fig Localization of potential protease cleavage sites and signals for N-glycosylation in the SARS S trimer. (A) The 3D structure of trimeric SARS S (amino acid residues: 261 to 1,058, protein structure ID: 5WRG, PMID: 28008928) was downloaded from the RCSB Protein Data Bank and analyzed using the YASARA software (www.yasara.org, PMID: 24996895). Since the deposited molecule contains an alanine at position 667 it was manually changed to arginine. Next, the molecular surface of SARS S was visualized and specific amino acid residues were highlighted as follows: Receptor binding domain, light blue; asparagine residues within N-glycosylation signals, blue; R667 and R797, red; T678, pink; K543/R544, green; R563/K566, yellow. (B) Individual inserts of the single amino acid residues investigated. N-glycosylation signals in proximity to the respective amino acid residues investigated are highlighted (white arrowheads).
(DOC)
