# Supplementary material for: Different residues in the SARS-CoV spike protein determine cleavage and activation by the host cell protease TMPRSS2
Source: PLoS One. 2017 Jun 21;12(6):e0179177. doi: 10.1371/journal.pone.0179177 (PMC5479546; doi:10.1371/journal.pone.0179177)
Supplement: S1 Fig — (A) The 3D structure of trimeric SARS S (amino acid residues: 261 to 1,058, protein structure ID: 5WRG, PMID: 28008928) was downloaded from the RCSB Protein Data Bank and analyzed using the YASARA software (www.yasara.org, PMID: 24996895). Since the deposited molecule contains an alanine at position 667 it was manually changed to arginine. Next, the molecular surface of SARS S was visualized and specific amino acid residues were highlighted as follows: Receptor binding domain, light blue; asparagine residues within N-glycosylation signals, blue; R667 and R797, red; T678, pink; K543/R544, green; R563/K566, yellow. (B) Individual inserts of the single amino acid residues investigated. N-glycosylation signals in proximity to the respective amino acid residues investigated are highlighted (white arrowheads). (DOC) [file pone.0179177.s001.doc]

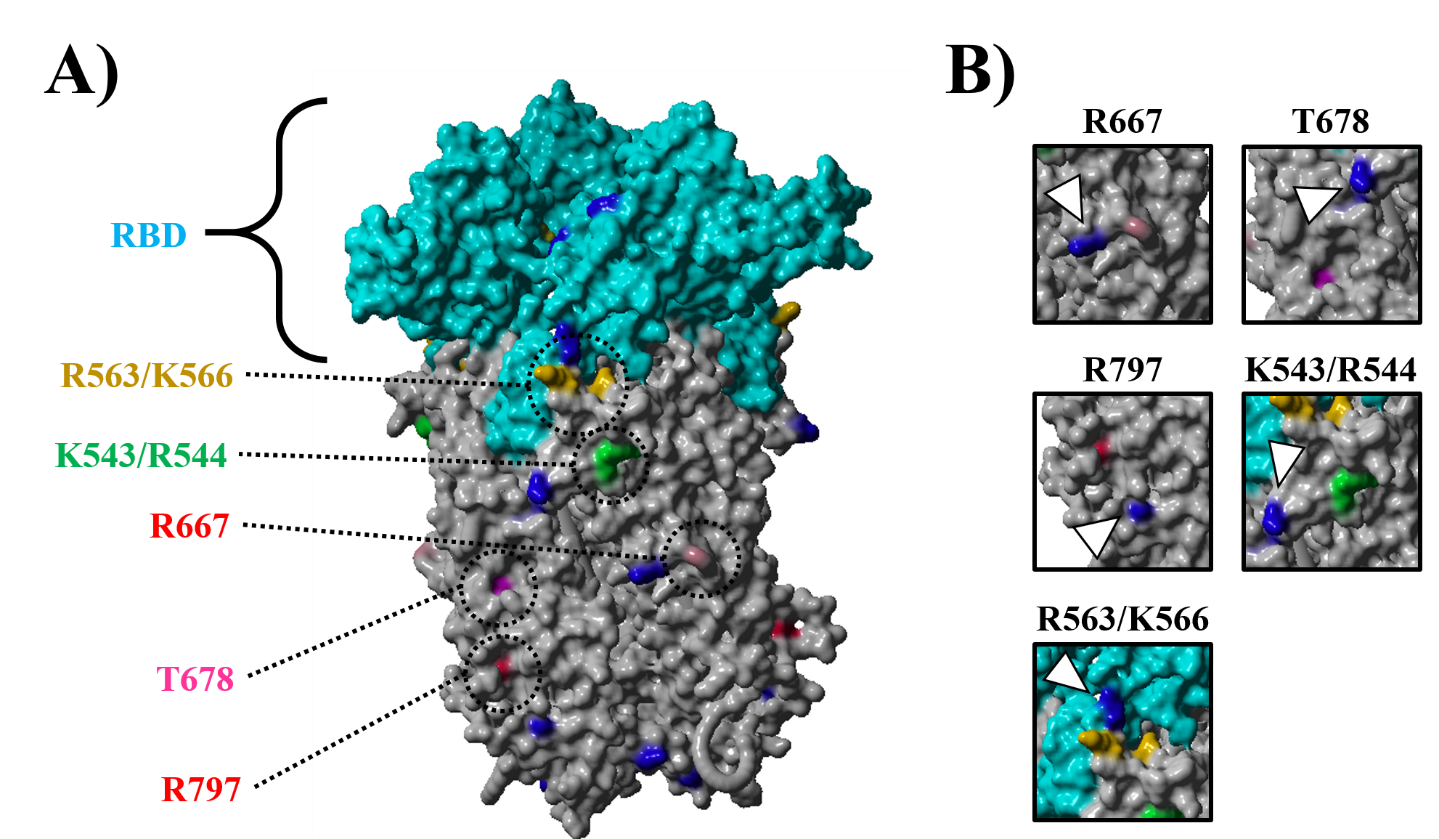
**S1 Figure Reinke *et al*.**

**S1 Fig. Localization of potential protease cleavage sites and signals for N-glycosylation in the SARS S trimer.** (A) The 3D structure of trimeric SARS S (amino acid residues: 261 to 1,058, protein structure ID: 5WRG, PMID: 28008928) was downloaded from the RCSB Protein Data Bank and analyzed using the YASARA software (www.yasara.org, PMID: 24996895). Since the deposited molecule contains an alanine at position 667 it was manually changed to arginine. Next, the molecular surface of SARS S was visualized and specific amino acid residues were highlighted as follows: Receptor binding domain, light blue; asparagine residues within N-glycosylation signals, blue; R667 and R797, red; T678, pink; K543/R544, green; R563/K566, yellow. (B) Individual inserts of the single amino acid residues investigated. N-glycosylation signals in proximity to the respective amino acid residues investigated are highlighted (white arrowheads).
